# Photoresponse from single upright-standing ZnO nanorods explored by photoconductive AFM

**DOI:** 10.3762/bjnano.4.21

**Published:** 2013-03-21

**Authors:** Igor Beinik, Markus Kratzer, Astrid Wachauer, Lin Wang, Yuri P Piryatinski, Gerhard Brauer, Xin Yi Chen, Yuk Fan Hsu, Aleksandra B Djurišić, Christian Teichert

**Affiliations:** 1 Institute of Physics, Montanuniversität Leoben, Austria; 2 Institute of Physics, National Academy of Sciences, Kiev, Ukraine; 3 Helmholtz-Zentrum Dresden-Rossendorf, Institut für Strahlenphysik, Dresden, Germany; 4 Department of Physics, University of Hong Kong, P.R. China

**Keywords:** AFM, nanorods, photoconductive AFM, photoconductivity, ZnO

## Abstract

**Background:** ZnO nanostructures are promising candidates for the development of novel electronic devices due to their unique electrical and optical properties. Here, photoconductive atomic force microscopy (PC-AFM) has been applied to investigate transient photoconductivity and photocurrent spectra of upright-standing ZnO nanorods (NRs). With a view to evaluate the electronic properties of the NRs and to get information on recombination kinetics, we have also performed time-resolved photoluminescence measurements macroscopically.

**Results:** Persistent photoconductivity from single ZnO NRs was observed for about 1800 s and was studied with the help of photocurrent spectroscopy, which was recorded locally. The photocurrent spectra recorded from single ZnO NRs revealed that the minimum photon energy sufficient for photocurrent excitation is 3.1 eV. This value is at least 100 meV lower than the band-gap energy determined from the photoluminescence experiments.

**Conclusion:** The obtained results suggest that the photoresponse in ZnO NRs under ambient conditions originates preferentially from photoexcitation of charge carriers localized at defect states and dominates over the oxygen photodesorption mechanism. Our findings are in agreement with previous theoretical predictions based on density functional theory calculations as well as with earlier experiments carried out at variable oxygen pressure.

## Introduction

One-dimensional ZnO nanostructures, so called ZnO nanorods (NRs), exhibit technological potential for many device applications. Having a wide band gap (3.37 eV at room temperature) and high exciton binding energy (60 meV) and being piezoelectric, ZnO is one of the most promising semiconductor materials. Fields of application include solar cells [1–4], piezo-actuators [5], energy harvesting devices [6], and photosensors [7–16].

A common feature of wide-band-gap semiconductors, such as ZnO, GaN, etc., is the presence of deep levels in the forbidden gap. The appearance of such levels as well as the density of electronic states associated with them depends on the number of defects within a semiconductor and is determined very often by the growth conditions [17–18]. The number of defects in ZnO is also known to be dependent on a post-growth sample treatment and even storage time [19], which may substantially alter its properties. Besides that, a diverse range of electronic properties appears in response to different surface conditions. The surface conductivity of ZnO is highly dependent on the presence of adsorbates [20–23]. Such surface defects serve as binding sites for chemisorption processes and may contribute to the scattering and trapping of carriers [24], which lower the intrinsic conductivity of the material. Moreover, the exposure of ZnO surfaces to light irradiation induces photodesorption of oxygen molecules from the surface [22], which leads in turn to a rise of conductivity. Therefore, the photoresponse in ZnO is very often considered as an exclusively surface-induced process whereas the role of the bulk properties is negligible. It has been shown recently that the phenomenon of persistent photoconductivity in ZnO can also be attributed to the presence of oxygen vacancies in the bulk [25]. Thus, more precise confirmation of the origin of this phenomenon is required and the characterization of single, separated as-grown ZnO NRs is of great importance.

In this work, we focus on the investigation of opto-electronic properties of single as-grown ZnO NRs by means of conductive force microscopy (C-AFM) and photoconductive atomic force microscopy (PC-AFM) techniques. C-AFM allows simultaneous but independent probing of the topography and electrical properties at the nanoscale [26] and is well suited to study the electric peculiarities of semiconductor nanostructures [27–29] and ZnO NRs in particular [30–31]. The capability of C-AFM to characterize local photocurrents has already been demonstrated more than a decade ago [32]. Recently, a similar approach of so called PC-AFM has been used to map local photocurrents in different types of solar cells [33–36] and to perform local, AFM-based photocurrent spectroscopy [37–38].

In contrast to the first setups where the sample was illuminated through the substrate [32–34,38], we use here a configuration of PC-AFM where the sample is illuminated from the top as already successfully applied to Si and SiGe nanostructures [39–40]. Such a scheme allows us to stay away from any limitations imposed by the substrate. Here, we employ this technique to study the electrical transport in individual upright standing ZnO NRs grown by thermal evaporation [41]. The results obtained together with those of time-resolved photoluminescence (PL) suggest that the photoresponse in ZnO NRs originates preferentially from the photoexcitation of charge carriers from defect-localized states. The experimental findings are in good agreement with previous theoretical predictions based on density functional theory calculations [42] and will be discussed on the basis of the presence of oxygen vacancies.

## Experimental

ZnO nanorods were grown by thermal evaporation of Zn in dry argon flow following a procedure described in more detail elsewhere [41]. The morphology of the resulting arrays of upright standing ZnO NRs was precharacterized by means of scanning electron microscopy (SEM) and intermittent contact mode AFM using PPP-NCHR™ probes from Nanosensors™ with a cantilever resonance frequency of approximately 330 kHz, a tip-curvature radius smaller than 10 nm, and a half-cone angle at the tip apex of about 10°. The applied forces have to be tuned carefully to avoid breaking the ZnO NRs [30,43]. For all experiments we used fresh probes as received from the provider without any additional treatment. Load-induced tip changes during the experiments could be ruled out by subsequent measurement of 2.5 nm high *para*-hexaphenyl islands grown on SiO_2_ as a reference [44].

The local photoelectric properties of the NRs were investigated using an MFP3D™ AFM from Asylum Research equipped with the standard ORCA™ module which allows one to carry out C-AFM experiments and to measure currents in the range of 1 pA to 20 nA. We also used an external amplifier (Model 1211 from DL Instruments) in order to extend the range of measured currents during the investigation of transient photoconductivity and photocurrent spectroscopy. Unlike the ORCA module, the setup with an external amplifier does not provide the possibility of voltage ramping. Therefore, the current-to-voltage curves were exclusively measured with the ORCA module in the limited range of ±20 nA. In order to carry out PC-AFM experiments, the setup was extended with an external illumination system, which consists of a 150 W Xenon lamp (white-light source) connected to an Omni-*λ* 150 monochromator (LOT-Oriel, wavelength continuously tunable from about 300 nm to over 1 μm), an optical fiber, and several collimating lenses. A calibration curve, which accounts for both the transmittance of the optical system and the emission spectrum of the light source, was recorded and used for the correction of the photocurrent spectra. The illumination from the AFM feedback laser diode, which has a wavelength of ≈850 nm, was also coupled to the illumination system. Nevertheless, the illumination at this wavelength does not affect the measurement since its wavelength lies in the region of transparency for ZnO.

We employed two different setups for measuring PC-AFM, which are described in the following. All measurements were carried out under ambient conditions on as-grown samples. The current–voltage (*I*–*V*) characteristics were recorded at the sample surface, which was under illumination directly from the optical fiber placed at an angle of about 15 to 20° with respect to the surface. For these measurements, we used a standard ORCA™ C-AFM holder and conductive diamond-coated DCP11™ probes from NT-MDT with a force constant of ≈5.5 N/m. The tip height for probes of this type is ca. 15–20 μm, which implies a restriction to the angle of illumination to the aforementioned 15–20° with respect to the sample surface due to shadowing. The illumination at large angle of incidence causes also an increase in the reflection from the surface, therefore the photoresponse is significantly suppressed in this configuration of illumination.

**Figure 1:**
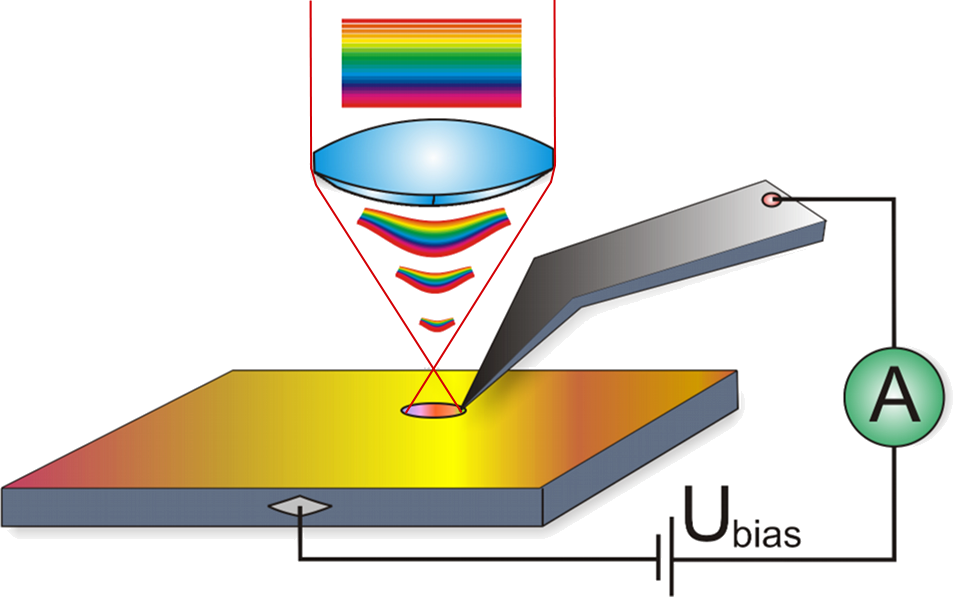
Schematic drawing of the PC-AFM setup. The sample in the present configuration was illuminated from the top side and biased. Use of ATEC-CONTPt™ probes helps to avoid shadowing by the cantilever.

For the investigation of transient photocurrent and photocurrent spectroscopy from single upright-standing ZnO NRs, the following procedure was employed. ZnO NRs were located in intermittent contact mode, and then the system was switched to contact mode AFM for the investigation of the transient photocurrent behavior of ZnO NRs. The loading force during the transient PC-AFM characterization was on the order of 20–30 nN, which is sufficient to establish a stable contact, but does not result in NR bending. For these experiments, we used Pt coated ATEC-CONTPt™ probes [45] with the force constant *k* = 0.02–0.75 N/m, and with the tip (tip-curvature radius smaller than 20 nm) located at the very end of the cantilever and visible from the top. As is depicted in Figure 1, the use of such conductive probes allows illumination of the ZnO NRs through the AFM’s optical system from the top.

To find the optimal probe location and to avoid possible probe damage, we performed imaging of the sample surface with the slow scan axis of the AFM switched off. After stable conditions were achieved, the conductive ATEC-CONTPt probe was located on the top facet of one of the upright-standing NRs, and the sample bias was applied. The photocurrent was recorded over a long period of time (ca. 3 h) at a sample bias of *U*_smp_ = −10 V. Such a high bias was applied to ensure a well detectable response. In order to determine the rise and decay time constants, we applied several cycles of illumination using white light (full spectrum) of the Xe lamp at 150 W.

The optical properties of ZnO NRs have been characterized macroscopically by means of time-resolved photoluminescence (TR-PL). The conventional steady-state PL and TR-PL were measured at 300 K. The monochromator used for both types of PL experiments has a linear dispersion of 0.8 nm/mm and was equipped with a photomultiplier tube as photodetector. The setup for the TR-PL experiments utilizes a stroboscopic oscilloscope with 0.1 ns gating registration system. As a source of optical excitation for PL measurements, we used the 337.1 nm emission line of a nitrogen laser with a pulse duration of 8 ns and a repetition rate of 100 Hz. PL emission was detected during the laser excitation pulse either at the leading edge of the laser excitation pulse (starting from ≈0.7 ns after the pulse onset), or at the trailing edge of the laser pulse after a variable delay time with respect to the onset of the laser pulse.

## Results

**Figure 2:**
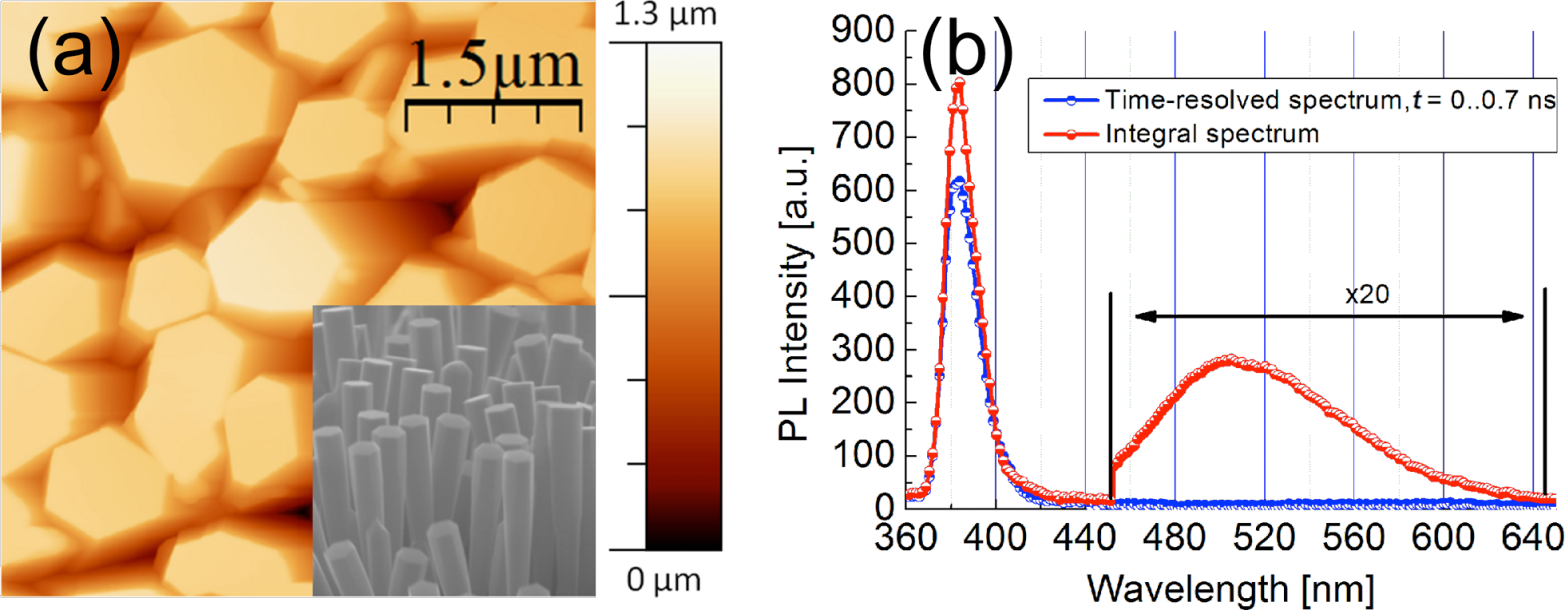
(a) 5 μm × 5 μm intermittent contact mode AFM image and SEM micrograph (inset) of ZnO nanorods grown by thermal evaporation, (b) time-resolved (blue curve) and integral (red curve) PL spectra from the array of upright standing ZnO nanorods, of photoluminescence obtained at 300 K.

As it is determined from the AFM image and the SEM micrograph presented in Figure 2a, the ZnO NR diameters vary in a wide range from 150 nm to 1.2 μm, whereas the rod lengths are ≈1.5 μm. Their cross-sectional shape is hexagonal; frequently it is even a regular hexagon. Their orientation is normal to the substrate surface with about 4% of the NRs deviating from the normal within about ±30°. Photoluminescence spectra of a ZnO NR array recorded using the 337 nm line of a pulsed nitrogen laser are presented in Figure 2b. The red curve shows the steady-state PL spectrum, each point on the plot averages the PL signal measured over approximately one second. The blue curve represents a TR-PL spectrum where each point was measured in a time frame from 0 to 0.7 ns from the moment of excitation.

**Figure 3:**
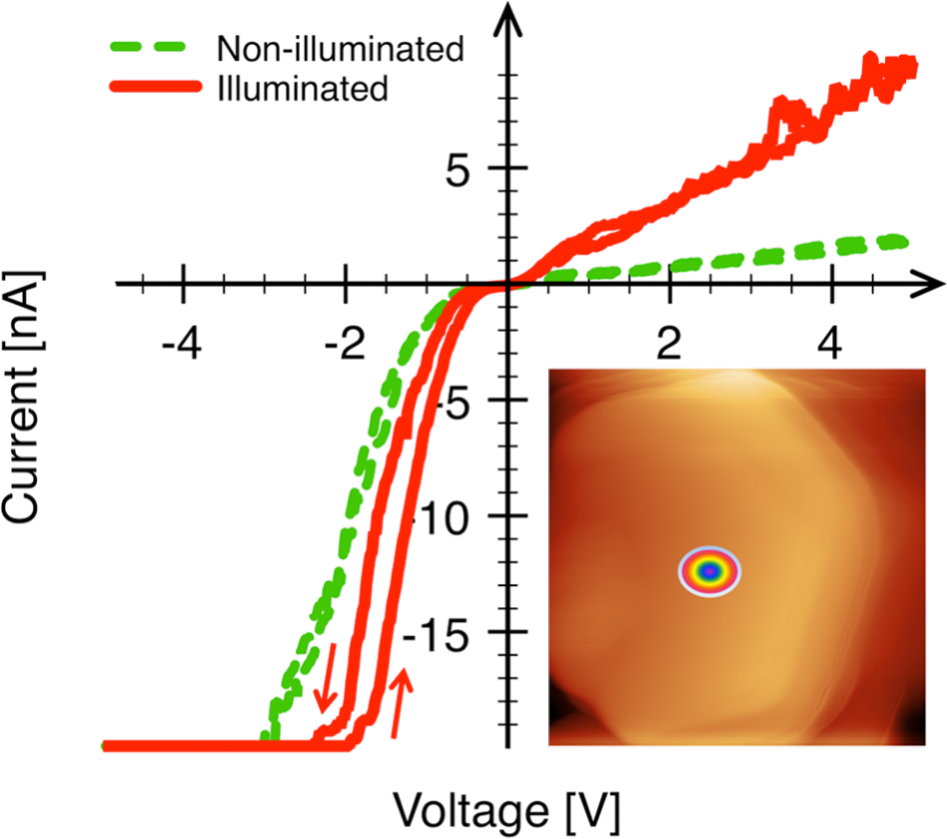
Current–voltage characteristics of dark (green curve, dashed) and illuminated state (red curve, solid) recorded from a single upright-standing ZnO nanorod by using a standard ±20 nA range amplifier. The illuminated characteristic was recorded by shining white light (150 W Xe lamp) at an angle of ca. 15–20° onto the sample. The arrows at the bottom indicate the direction of the voltage ramp.

Figure 3 shows the influence of illumination on the *I*–*V* characteristics of a single upright-standing ZnO NR. The dark and illuminated *I*–*V* characteristics were both recorded with 30 s delay between the measurements, the voltage was applied to the substrate and ramped forth and back with a rate of 20 V/s. The *I*–*V* curve was first measured in the dark, then under illumination. A repetition of this sequence after an additional delay of ca. 10 min did not yield any changes in the characteristics.

Both, illuminated and dark characteristics demonstrate rectifying behavior with a rather significant deviation of the reverse current from the zero level. Moreover, the reverse current in both cases demonstrates a linear dependence on the applied voltage, which can be attributed to the photoexcitation of charge carriers from the valence band to a defect-perturbed host state (PHS, depicted as transition (3) in Figure 6, see below). The *I*–*V* characteristics recorded from ZnO NRs under illumination are degenerated with high currents at reverse (positive) sample bias, which indicates an increase of the charge-carrier concentration. The rectifying *I*–*V* characteristics are associated with the Schottky contact between AFM tip and ZnO NR. The Schottky barrier heights (SBHs) were estimated, using the same method as applied in [31], to be 0.22 ± 0.06 eV for the dark and 0.18 ± 0.06 eV for the illuminated case. The corresponding ideality factor was ≈2.2 in both cases.

**Figure 4:**
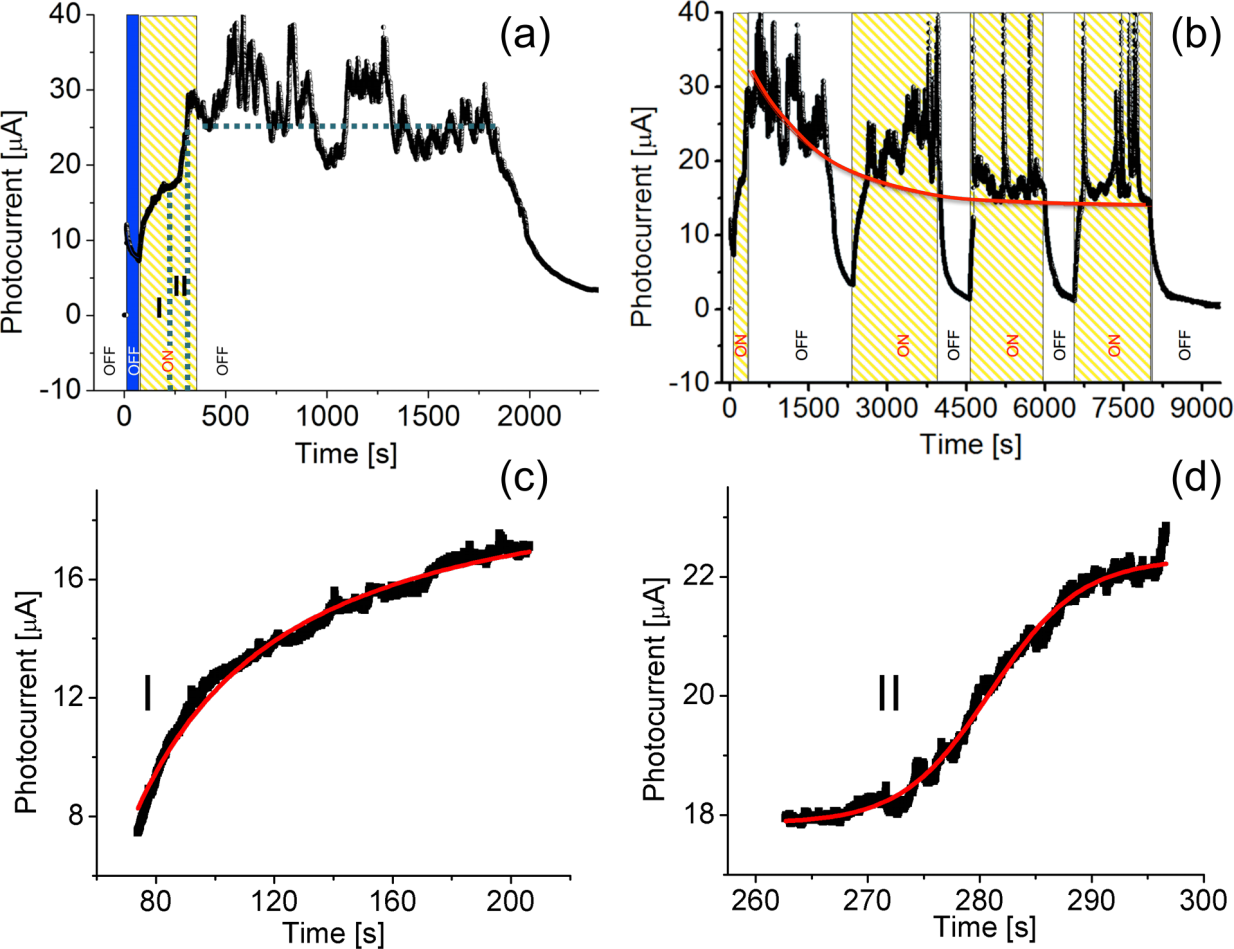
(a) Photocurrent rise and relaxation during the first cycle of the experiment. The bias of −10 V was applied at 0 s, the blue shaded area indicates the initial dark current decay. After the illumination was switched on the photocurrent rose in two steps, which are separated on the figure by vertical dashed lines and marked as I and II. (b) Several cycles of the illumination; the sample bias is −10 V, the source of illumination is a 150 W Xe lamp (white-light source). The periods under illumination are marked by the yellow shaded areas. (c), (d) First two steps of the photocurrent increase, marked as I and II in (a), fitted by Equation 2 (red curves).

In Figure 4, we present the PC-AFM results for a single ZnO NR under illumination from the top using the setup shown in Figure 1. Here, a bias of *U*_smp_ = −10 V has been applied to the substrate and the current was measured as a function of time in the dark and under illumination. For illumination, the full spectrum of the Xe-lamp was used. The transient photocurrent curve recorded during the first cycle of the illumination is shown in Figure 4a. The bias was applied at time 0, which caused a current jump from 0 to 12 mA, followed by an exponential current decrease (blue shaded area in Figure 4a), which can be fitted well by:

[1]
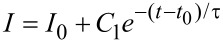


where *t* is time, *τ* is the decay time constant, *t*_0_ is the decay time offset, *I*_0_ and *C*_1_ are the offset and amplitude of the current decay, respectively. The time constant of the dark current decay after the initial application of sample bias was determined to be *τ* ≈ 18 s.

The illumination of the sample surface for the first cycle was started at *t* = 75 s and stopped at *t* = 300 s when the current was saturated. After the illumination was switched on, the photocurrent raised in two steps. Each step of this raise is best approximated by the so called logistic equation:

[2]
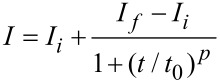


where *p* is a number that can take various real values, increasing with *p* = 1.5 for the first step, *t*_0_ is the rise-time offset, and *I_i_* and *I_f_* are the initial and final current levels. These first two steps of the photocurrent increase marked as I and II in Figure 4a fitted by Equation 2 (red curves) are presented in Figure 4c and Figure 4d, respectively. Surprisingly, the photocurrent persisted at about the level of saturation from *t* = 500 s to *t* = 1835 s (marked by the horizontal dashed line in Figure 4a). There are instabilities in the form of pronounced current spikes and slumps, which appear randomly and originate likely from mechanical instabilities in the tip-to-sample contact. At *t* = 1835 s the current abruptly decreases, which again can be well fitted by Equation 1. In this case the best fit yields a decay time constant of *τ* ≈ 163 s.

Figure 4b shows in addition to the first cycle further cycles of illumination of the sample and corresponding transient photocurrent behavior. During all cycles, excluding the first one, the photocurrent raised, reached the saturation level, and stayed there until the illumination was switched off. The exponential current decrease in each cycle is well fitted by Equation 1 resulting in an average decay time constant of *τ* = 140 ± 20 s, and the corresponding average raise time constant, also determined from Equation 1, is equal to *τ* = 20 ± 9 s. The slow return of the dark current value after illumination, known as persistent photoconductivity, has frequently been reported for ZnO material [22,46]. Interestingly, the saturation level is decreasing in time. When fitted, by using again Equation 1 (red curve in Figure 4b), it gives a decay time constant of *τ* ≈ 1400 s for the saturation level. The transient photocurrent experiments performed on a single separated NR provide information about the characteristic rise and decay time of the photoresponse. An interesting peculiarity of the transient photocurrent measured at different ZnO NRs is the already mentioned observation that the current initially stays at the saturation level during the first cycle of the illumination, even when illumination is switched off, and then rapidly decays. In principle, this behavior can be caused by several mechanisms, such as water dissociation and desorption, recharging of defects, etc. We believe that this phenomenon should be considered as a current-induced electrochemical process as we will argue in the discussion.

**Figure 5:**
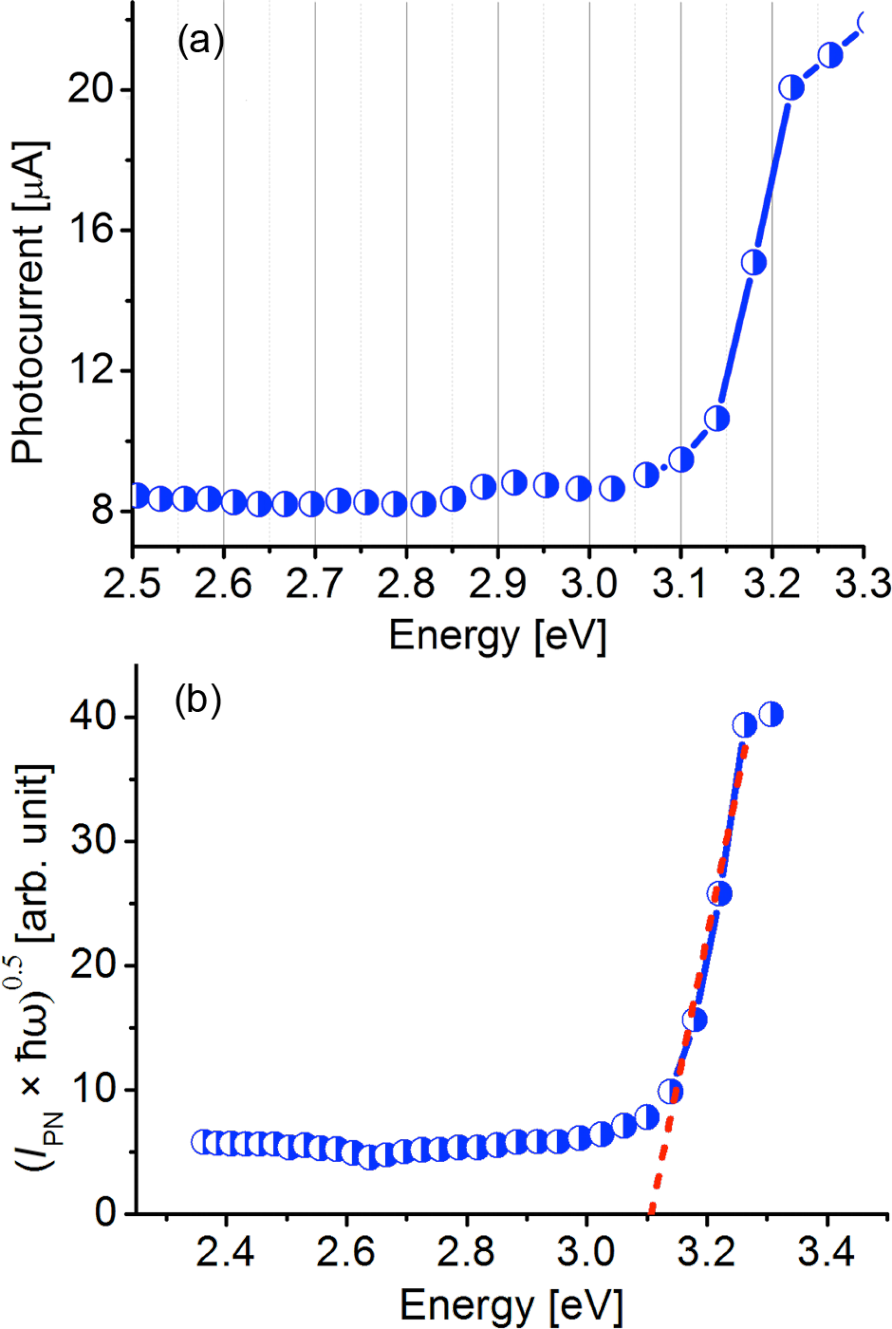
(a) The photoconductivity spectral response from a single upright standing ZnO NR recorded using a 150 W Xe lamp connected to the monochromator. The sample bias was −10 V. (b) The quantum efficiency of light conversion linearized in coordinates 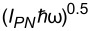 versus 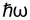. The dashed line represents extrapolation of the linearized region until the intersection with the x-axis.

In order to gain insight into the electronic structure of single upright standing ZnO NRs, we measured the spectral characteristics of the photocurrent by means of PC-AFM. The result of these measurements at a sample bias of −10 V is presented in Figure 5. The photocurrent spectra were measured starting from longer wavelengths with a time delay varying in the range from 30 s to 2 min between acquisition of two neighboring data points. This time delay was sufficient to account for the time needed for photocurrent saturation. Each data point in the photocurrent spectrum represents the average value of 100 points measured at 1 kHz acquisition rate. In addition to the measured data (Figure 5a), we present also the extrapolation of the normalized spectrum to *I_PN_* · 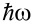 = 0 (Figure 5b). The latter provides the energy corresponding to the transition involved in the photocarrier generation process. Interestingly, it has been found that the NRs are already sensitive to illumination with a wavelength of 400 nm, i.e., with a corresponding photon energy of 3.1 eV which is smaller than the band gap of ZnO (3.37 eV).

## Discussion

The standard model to explain the photoresponse in ZnO involves the photodesorption of oxygen molecules [22]. Adsorption of oxygen on the ZnO surface causes the capture of electrons leading to the formation of a negatively charged layer and a depletion region near the surface. The presence of adsorbed oxygen introduces a trap surface state within the band gap. The probability of population of this state drops with an increasing amount of oxygen adsorbed on the surface. As a result, the process of electron transport from the bulk to the surface is also becoming slower. The level of saturation is achieved when the energy of the trap state reaches the Fermi level due to the band bending. It is generally assumed that when the surface is exposed to light with photon energies higher than the band gap in ZnO, electron–hole pairs are created [22]. Then, the holes move toward the surface in the electric field of the surface depletion region and recombine with the electrons there 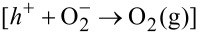. This results in an excess of electrons, which were generated by the light absorption contributing to the photocurrent when the sample is biased. This model implies also that the photoconductivity in ZnO is limited to the fundamental absorption range (i.e., for photon energies higher than the band gap). For photon energies smaller than the band gap, the conductivity could increase only at the expense of photoexcited electrons from defect levels. This process excludes the generation of free holes and photodesorption due to the recombination of free holes with trapped electrons on the oxygen molecules. Moreover, the slow kinetics of the photocurrent decay, even under high oxygen partial pressures, is not explained satisfactorily by this model. The photocurrent spectrum presented in Figure 5a reveals that the sample becomes photosensitive at ≈400 nm (3.1 eV). To estimate a band-gap energy that could be compared with the values derived from the PL experiments, we normalized the data presented in Figure 5a taking into account the emission spectrum of our light source (i.e., we switched to the presentation of the characteristic of quantum efficiency of light conversion). A linearization of the data by plotting in coordinates 
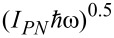
 versus 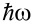, where the *I_PN_* is the normalized photocurrent for incident photons of energy 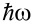, is presented in Figure 5b. An extrapolation of the linear region marked by the dashed line in Figure 5b yields the transition energies involved in the photocarrier generation process. The obtained value *E*_min_ ≈ 3.1 eV is the minimum photon energy sufficient for the photoexcitation of mobile charge carriers.

The value of 3.1 eV turned out to be at least 100 meV lower than the band gap energy determined from the PL experiments as will be discussed in the following. The TR-PL spectra presented in Figure 2b reveal both band-edge and defect emission. There a strong peak at 383 nm (3.2 eV), which occurs within 0.7 ns after onset of the excitation pulse can be attributed to the emission of localized excitons. Therefore, the absorption of light with photon energies of 3.2 eV and higher may lead to the generation of excitons with possible subsequent dissociation into free charge carriers. The broad band visible in Figure 2b (integral spectrum) with the maximum at 504 nm is commonly attributed to the presence of oxygen vacancies [17]. The crystalline quality can be estimated by a simple comparison of the intensities of the exciton peak and defect band. From the PL measurements we conclude that the generation of electron–hole pairs at room temperature becomes possible with a photon energy 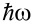 ≥ 3.2 eV under the formation of free excitons, indicative of the possible presence of oxygen vacancies. Based on the latter findings, we conclude that the experimentally observed photocurrent cannot be simply explained by band-to-band transition with subsequent electron–hole pair generation and oxygen desorption, as assumed in the standard model [22].

**Figure 6:**
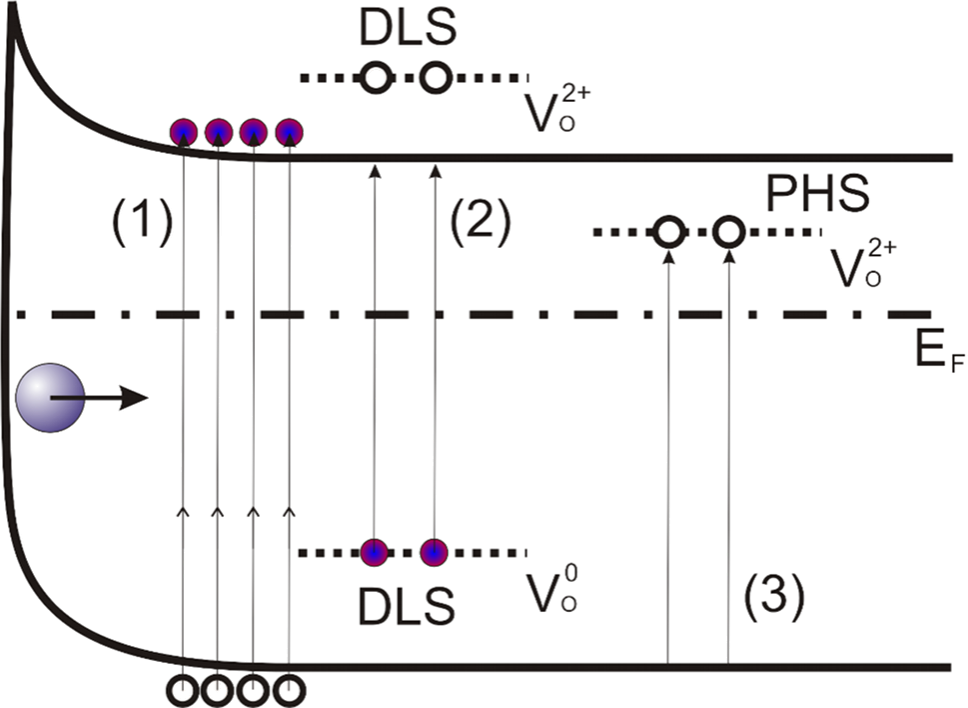
Schematic energy-level diagram of ZnO taking into account the existence of theoretically predicted [42] α- and β-type configurations of oxygen vacancies. The processes of electron–hole pair generation via band-to-band absorption, charge carrier photoexcitation from a defect localized state (DLS) and to a perturbed host state (PHS) of an oxygen vacancy are marked as (1), (2) and (3), respectively.

A theoretical explanation of the persistent photoconductivity in ZnO, which also explains the lowering of the minimum photon energy, has been provided by Lany and Zunger on the basis of density functional theory calculations [42]. The corresponding energy-level diagram also accounting for the band bending due to surface states is presented in Figure 6. The model involves a two-step process where an oxygen vacancy V_O_ changes its state from nonconductive (α-configuration of defect localized state (DLS), 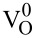) to conductive (β-configuration of DLS, 
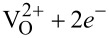
) with the subsequent appearance of a PHS below the conduction band minimum under illumination. Recently, this model was also supported by experiments carried out under different oxygen levels [10]. The electrons from the energy level that corresponds to the α-configuration are photoexcited to the conduction band contributing to the photocurrent. The existence of the PHS state implies also a trapping of the mobile charge carriers in this state. The transition back to the nonconductive state requires a simultaneous thermal activation of the electrons from the PHS state to the conduction band and capturing them back at the defect. Both conditions, thermal excitation from the PHS and capture by the DLS, have to be fulfilled simultaneously, which implies slow kinetics for the photocurrent decay. The presence of the PHS state makes a transition from the valence band to the PHS state possible, which leads to the formation of holes and therefore to an increase in p-type conductivity under illumination. In fact, both experimental observations, i.e., the photoresponse for illumination at wavelengths from 400 nm on (Figure 5) as well as the increased p-type conductivity upon light exposure (Figure 3), are consistent with the model of Lany and Zunger provided the presence of defect states around 100 meV above the ZnO valence band edge.

Indeed it has been demonstrated and discussed in a recent paper [47] that the difference between the nominal band gap *E*_g_ = 3.37 eV at 300 K known for ZnO and the optically determined band gap values 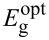, being at least 100 meV smaller, indicates the presence of optically active defects in all ZnO samples investigated. This means that the corresponding defects are characterized by this energetic amount from either the valence or conduction band within the band gap. The true nature of these defects still has to be determined and may depend on the method of crystal growth. Thereby, an involvement of hydrogen cannot be excluded and should at least be considered as well [43,47].

A further point to discuss is the observed transient current behavior. Especially, the deviation of the current evolution during the first cycle of illumination compared to the successive illumination cycles is puzzling. When the illumination was switched off after the initial light exposure, the current remained fluctuating around the level of saturation (marked by the dashed line in Figure 4a) for about 21.6 min and then dropped exponentially. Although we observe this behavior reproducibly for different NRs, we can still only speculate on the origin of this phenomenon. In fact several contributions may occur. Initially, the ZnO surface under the AFM tip is covered with adsorbates such as water and oxygen. Under the applied conditions, the water layer may be several monolayers thick. Once the current rises the initial surface conditions will be changed due to electrochemical processes, electromigration and local power dissipation. One of the major changes will be a reduction of the water present due to thermal desorption. On the one hand, this changes the contact properties significantly and on the other hand, the contact becomes more stable. Obviously, this situation occurs only in the first cycle. For all successive illumination cycles the exponential current decrease set in immediately when the light was switched off. Additionally, a decrease of the photocurrent saturation level with an increasing number of illumination cycles is observed. Such a behavior might be explained by different mechanisms. The pronounced current fluctuations around the current saturation level are most likely caused by contact instabilities, even though the experiments were carried out in a regime where the tip loading force should be sufficient to provide a stable contact.

It should also be noted that the current level is in the μA range, which leads to a considerable power dissipation and heating of the contact between AFM tip and NR. For the applied contact forces, the effective contact radius between AFM tip and ZnO NR can be estimated to be *r*_C_ ≈ 1.6 nm [48–53]. Assuming that most of the applied potential drops across the AFM-tip–NR contact, the average dissipated power at the contact is *P* = *U* · *I* ≈ 0.25 mW. Since the effective contact radius is small compared to the radius of curvature of the AFM tip (≈20 nm), the contact region may be modeled as a flat disc with diameter 2*r*_C_ and the NR as a semi-infinite solid (NR diameter >> 2*r*_C_). For this case, Δ*T* ≈ 0.5 · *P*/(*k*_th_ · 4*r*_C_) = 195 K [54], with the thermal conductivity of ZnO, *k*_th_(ZnO) ≈ 100 W·m^−1^·K^−1^ [51]. The factor 0.5 was introduced to take into account that a part of the energy dissipated at the contact is conducted via the tip. This means that local NR temperatures of at least 495 K can be expected. Further evidence for a pronounced temperature increase at the contact point can be found by comparing the SBHs obtained in a previous study [31]. There, an amplifier with higher gain, allowing lower currents, was used. The SBH between a diamond coated tip and the ZnO NR was determined to be 0.54 eV. The large difference between the 0.22 eV found here and the 0.54 eV can be simply explained by a higher junction temperature induced by the higher current. On the one hand, this “heating” affects the surface properties due to increased desorption, and on the other hand it might lead to a local annealing of the nanorod. Since the resulting annealing takes place in an oxygen-rich environment, a decrease of the ZnO oxygen vacancies near the contact region is possible. It has been shown recently that annealing at temperatures below 700 K already has an effect on the structural and optical properties of ZnO [55–56]. This could be responsible for the quenching of the saturation current with increasing number of illumination repetitions (indicated by the red curve in Figure 4b). Within the size range of the NRs investigated here, no indication of a size-dependent photoresponse was noticed. A systematic study on the size dependence has not been performed yet and is a topic for future investigations.

## Conclusion

A novel PC-AFM technique enabling sample illumination from the top has been implemented to study the optoelectronic properties of individual upright-standing ZnO nanorods under ambient conditions. The corresponding photocurrent spectrum revealed that the minimum photon energy sufficient for photocurrent excitation is 3.1 eV. This value turns out to be at least 100 meV lower than the band-gap energy determined from macroscopic photoluminescence experiments. This is inconsistent with the frequently applied model for ZnO photoconductivity involving electron–hole pair generation by light induced band-to-band excitation and subsequent oxygen desorption as a surface process [22]. We suggest instead that the observed peculiarities of photoconductivity under ambient conditions can be attributed to the presence of defect states in the band gap. In fact, our findings support theoretical predictions based on density functional theory calculations [42], which state the presence of oxygen defect states above the valence band edge. Our observations also agree well with earlier experiments carried out at variable oxygen pressure [10]. The observed transient photocurrent may also be related to the local annealing of oxygen defects due to power-dissipation heating at the nanocontact formed between the ZnO NR and the conductive AFM tip.
